# Tetrabutylphosphonium Bromide Reduces Size and Polydispersity Index of Tat_2_:siRNA Nano-Complexes for Triticale RNAi

**DOI:** 10.3389/fmolb.2017.00030

**Published:** 2017-05-16

**Authors:** Jordan T. Pepper, Priti Maheshwari, Alicja Ziemienowicz, Paul Hazendonk, Igor Kovalchuk, François Eudes

**Affiliations:** ^1^Lethbridge Research Centre, Agriculture and Agri-Food CanadaLethbridge, AB, Canada; ^2^Department of Chemistry and Biochemistry, University of LethbridgeLethbridge, AB, Canada

**Keywords:** Cell-penetrating peptides, dynamic light scattering, phytoene desaturase, RNAi, tetrabutylphosphonium bromide, triticale

## Abstract

Cell-penetrating peptides (CPPs) are short 8–30 amino-acid oligopeptides that act as effective transducers of macromolecular cargo, particularly nucleic acids. They have been implemented in delivering dsDNA, ssDNA, and dsRNA into animal and plant cells. CPPs and nucleic acids form nano-complexes that are often 100–300 nm in size but still effectively transit the cell membrane of animal cells, but are less effective with plant cells due to the plant cell wall. To overcome this obstacle, nano-complexes of the CPP Tat_2_ and various lengths of nucleic acid (21-mer siRNA duplex (dsRNA) to ~5.5 kb circular plasmid) were evaluated for size using dynamic light scattering (DLS), under conditions of increasing ionic strength (I_c_) and addition of phase transfer catalyst salts (tetrabutylammonium bromide-TBAB and tetrabutylphosphonium bromide-TBPB) and sugars (maltose-mannitol solution). It was found that the combination of 21-mer siRNA:Tat_2_ complexes with TBPB produced small 10–20 nm diameter nano-complexes with a polydispersity index (PDI) of ~0.1. Furthermore, it was found that for each length of nucleic acid that a linear mathematical relationship existed between the theoretical volume of the nano-complex and the nucleic acid length. Next, nano-complex formulation was tested for its ability to carry small interfering RNA molecules into plant cells and to trigger silencing of phytoene desaturase (PDS) in Triticale leaves. RT-qPCR showed 75% suppression of PDS, demonstrating that TBPB acts as an adjuvant in effecting the entry and efficacy of siRNA in young Triticale plants.

## Introduction

Cell-penetrating peptides (CPPs) are non-viral delivery vehicles mostly derived from the transduction domains of proteins, usually consisting of 8–30 amino-acids. Often, they carry a net cationic charge under physiological pH originating from their constituent basic residues such as arginine and lysine (Brock, 2014). These peptides have been used for the delivery of negatively charged nucleic acids into both plant and animal cells due to their ability to traverse the cell membrane. The delivery of plasmid DNA, dsDNA, ssDNA, and dsRNA (e.g., siRNA) using CPPs has been successful in various plant and animal cell-culture systems (Chugh and Eudes, 2008; Liou et al., 2012). The success of these systems, however, has been based heavily on the physicochemical characteristics of the nano-complexes formed between the nucleic acids and the CPPs. The three principle characteristics that apparently govern the transfection efficiency of these nano-complexes are size, polydispersity, and zeta-potential (Cardoso et al., 2013; Liu et al., 2013).

To address these particular parameters, the standard analysis tool is dynamic light scattering (DLS). It is a photometric technique that analyzes the Rayleigh scattering of monochromatic laser light by colloidal particles in a dispersion medium (Jafari et al., 2014). This may be used to determine size distribution via the Stokes-Einstein equation. The zeta-potential is obtained using a similar method (often performed by the same DLS machine) called electrophoretic light scattering (ELS) that converts electrophoretic mobility to zeta-potential. In this study, DLS and ELS have been used to characterize and optimize the conditions for formation of nano-complexes for delivery into plant cell culture systems. Since the conditions that affect nano-complex formation are multitudinous, the purpose of this study was to explore and discover conditions that minimize size and polydispersity index (PDI), as well as modulate zeta-potential.

The mechanism of entry of CPPs into the cell has remained a subject of much controversy due to conflicting information from a number of reputable studies (Bechara and Sagan, 2013). It is generally agreed that the process of CPP complex uptake is manifest either through direct transport, using interactions with the cell membrane phospholipids, or through endocytosis (Ziegler and Seelig, 2011). In the case of the latter, the process must be 2-fold; endosome formation followed by endosomal release. Since both mechanisms require modulation of the cell membrane, the use of phase catalyst salts in conjunction with CPPs presents as a possible method of increasing infiltration of CPP nano-complexes across the cell membrane. Phase catalyst salts may help increase cell membrane infiltration by assisting in shuttling negatively charged ions (e.g., nucleic acid) into non-polar conditions similar to the hydrophobic core of the cell membrane. For this purpose, the formation of stable ternary complexes of CPPs, nucleic acids and tetrabutylammonium bromide (TBAB) or tetrabutylphosphonium bromide (TBPB), was explored. The cationic components of these salts have already been shown to complex with DNA, and are also documented phase-catalysts, binding and moving anions from aqueous hydrophilic environments to non-aqueous hydrophobic environments (Di Profio et al., 2010). Polymers with tertiary ammoniums and phosphoniums have been utilized as well for delivery of nucleic acid cargoes in animal cells (Ornelas-Megiatto et al., 2012; Loczenski Rose et al., 2015), and phosphonium salts have been shown to have mitochondrial targeting properties (Muratovska et al., 2001).

Most of the work performed with CPPs has focused on delivery in animal cells, mammalian cells in particular, for which high efficiency delivery has been demonstrated using a number of cargoes covalently or non-covalently bound to CPPs. The same cannot be said *in planta* due to factors that frustrate uptake, such as, the cell wall impermeability, differential rates of macropinocytosis and differences in membrane physicochemistry. One particular difference not previously investigated is the effect of plant tissue culture media composition on complex formation and its subsequent stability. Due to the preponderance of animal cell culture systems in research platforms for CPP investigation, the medium of interest was typically either PBS or serum based media (Hemp et al., 2012b; Jafari et al., 2014). As a result, very little information gathered from other complex formation studies is applicable to plant tissue culture systems that involve use of medium with high sugar content.

Tat_2_ (RKKRRQRRRRKKRRQRRR) was evaluated in terms of its complexation with 21-mer dsRNA (21-mer siRNA duplex), 0.5, 1, and 3 kb dsDNA as well as a circular plasmid (5,556 bp), either in the presence of TBAB, TBPB or CaCl_2_. Some work was completed with regards to experimenting in delivering TBPB modified siRNA:Tat_2_ complexes to triticale leaf tissue to induce successful silencing of the phytoene desaturase by the RNA interference (RNAi) mechanism. RNAi is expedited by small molecules (siRNAs; ~21–24 nt long) that interferes with messenger RNA (mRNA), resulting in reduced protein synthesis.

## Materials and methods

### Nucleic acid preparations

The 1 kb ladder from Invitrogen was used as the source of all linear dsDNA fragments. Ladder was prepared according to manufacturer instructions and run on a 1.0% agarose gel stained with Gel Red (0.5x) for 1 h in 1xTAE buffer at 90 V. The desired linear dsDNA fragments (0.5, 1, and 3 kb) were visualized using an ultraviolet light and extracted from the gel using a scalpel. The DNA fragments were then purified from the agarose using the Qiagen gel extraction kit, following the manufacturer instructions, eluting all final fragments from columns using Ultrapure Water (Sigma-Aldrich). Isolated DNA stocks were diluted to 20 ng/μl.

The pMS plasmid with a segment of 3,033 bp inserted at the multiclonal site (total length 5,556 bp), was used for study of pDNA size and PDI in complex with Tat_2_. The plasmid was cloned using *E. coli*, strain DH5α. *E. coli* containing pMS were taken from a glycerol stock and cultured overnight in 200 ml of LB media and purified from cells using a plasmid maxiprep kit according to the manufacturers instructions (Sigma-Aldrich). Plasmid was eluted from the included columns using Ultrapure water (Sigma) and diluted to 20 ng/μl for use in complex formation.

The 21-mer siRNA duplex was purchased from IDT as the sequence, 5′-AUGGAGACGCCGUCGUU-3′ and its complementary strand 5′-CGAGGACGGCGUCUCCAUGUU-3′ producing a duplex (molecular weight of 13,368.1 Da) with double U overhangs. The lyophilized product was dissolved in Ultrapure Water and subsequently diluted to a stock concentration of 2.0 μM (26.7 ng/μl). Final concentrations of all nucleic acids were determined by NanoDrop 8000 UV-Vis spectrophotometer (Thermo Scientific).

### Nano-complex sample preparation and size analysis

Sample preparation and analysis methods were based on a previous work involving siRNA nano-complexes (Jafari et al., 2014). Individual samples were prepared in a PCR well plate. Maltose-mannitol (MM, 90 mg/ml maltose, 9 mg/ml mannitol) solutions with varying amounts of CaCl_2_ (0.00, 78.43, 156.86, 235.29, 313.73, and 392.16 mM) were prepared in ddH_2_O at pH 7.0 and filtered twice through 0.2 μm cellulose syringe filters (VWR) using a 5 ml syringe. From these stock solutions 17 μL were aliquoted into well plates for each CaCl_2_ concentration and 1 μl of stock dsRNA or DNA was added. Finally, 1 μl of the appropriate amount of Tat_2_ peptide (varied by N:P ratio) was added and 1 μl of MM solution with no CaCl_2_ was added to bring the final volume to 20 μl The N:P ratio for Tat_2_ to nucleic acid was defined as the ratio of cationic lysine and arginine residues of Tat_2_ (N) to the number of nucleic acid backbone phosphates (P). The N:P ratios used were 1:1, 4:1, and 8:1 for dsRNA experiments and only the 8:1 N:P ratio was used for experiments with DNA. All these preparations resulted in 20 μl samples with final CaCl_2_ concentrations of 0.00, 66.66, 133.3, 200.0, 266.6, and 333.3 mM, corresponding to ionic strengths (I_c_) of 0, 0.2, 0.4, 0.6, 0.8, and 1.0 M respectively. The reaction mixtures were allowed to incubate for 15 min before measurement.

In the case of formulations containing tetrabutylammonium bromide (TBAB, Sigma) or tetrabutylphosphonium bromide (TBPB, Sigma), instead of MM, 1 μl of 8.4 nmol/μl TBAB, or TBPB dissolved in MM was added after 10 min of incubation time between the nucleic acid and Tat_2_ and allowed a further 5 min of incubation time. This was a 100:1 molar charge ratio of N^+^ or P^+^ core atoms of each salt cation to backbone phosphates (P). This ratio was kept stationary for all experiments involving TBPB or TBAB. The final concentrations of both TBAB and TBPB were 420 μM, contributing little to the ionic strength of solution. Experiments with TBPB and TBAB were performed for dsRNA formulations and only experiments involving TBPB were performed with DNA formulations.

Size analysis was carried out immediately after incubation in a wetted (with 200 μl MM solution) low volume quartz sizing cuvette (ZEN 2112). The entire sample was loaded into the cuvette and measured on a Zetasizer Nano ZS (Malvern) with a 633 nm laser at 173° backscatter. Data was analyzed using the CONTIN algorithm in the Zetasizer software v.7.02 (Malvern). Three repeat measurements were made of each sample and means and standard deviations of both the primary particle size distribution peak, and PDI were calculated and plotted.

### Advanced size analysis

Hydrodynamic diameters (mean measured sizes, D_H_) from formulations which resulted in minimally sized nano-complexes for each size of nucleic acid were used to calculate hydrodynamic volumes (V_H_) under the assumption of a spheroid for all nano-complexes. V_H_ was plotted as a function of the approximate length of the nucleic acid based on the assumption of 0.33 nm/bp. In the case of circular plasmid the length was halved to account for supercoiling and circular shape. Linear regression was used to determine a mathematical relationship between nucleic acid length and theoretical nano-complex volume, and the slope was used to determine the accuracy of fit of each individual data point.

### Zeta-potential analysis

Zeta-potentials (symbol-ζ) were analyzed for circular plasmid, and for 21-mer siRNA in formulations as previously described but with an increase in the concentration of nucleic acid and Tat_2_ by five-fold (5 ng/μl, nucleic acid). This was done at a 1:1, 4:1, and 8:1 N:P ratio for each with and without TBPB (100:1 molar charge ratio as previously described), as well as nucleic acid alone. Zeta-potential of samples were read using a capillary zeta-cuvette with gold electrodes in a Zetasizer-Nano (Malvern). The samples were prepared to a volume of 700 μl to fill the cuvette. Zeta-potential measurements were recorded and displayed to a number of significant figures provided by and to the accuracy of the Zetasizer-Nano instrument.

### Delivery of siRNA in young triticale leaf tissue

Seeds of *Triticosecale sp. Whittmack cv* Sunray, were planted in 8 × 4 root trainers and allowed to grow in a growth cabinet at 12–15°C, and a photoperiod of 19 h/day at an intensity of 300 μE/m^2^/s. At 13 days post planting, at the two leaf stage, plants were inoculated using a 1 ml sterile syringe (VWR) a formulation of one of the following in either ddH_2_O or maltose-mannitol solution; solution only (Control), TBPB only, siRNA only, Tat_2_ only, TBPB+Tat_2_, TBPB:siRNA, siRNA+Tat_2_, Tat_2_:siRNA:TBPB, scsiRNA (scrambled siRNA), and Tat_2_:scsiRNA:TBPB. Together there were a total of 18 treatments performed in a minimum of four replicates, where a single repetition consisted of 18 individual plants. A total of 250 pmol of siRNA in 100 μl was injected in all siRNA treatments, with TBPB, and Tat_2_ being added proportional to the siRNA at the 8:1 N:P ratio of Tat_2_ to siRNA and TBPB at the 100:1 ratio of positive to negative ratio of phosphonium ions to phosphates. The injections were directed toward the base of the youngest leaf after which the plants were returned to the growth cabinet (12–15°C, photoperiod of 19 h/day at an intensity of 300 μE/m^2^/s) until sample collection 24 h later.

The 21-mer siRNA duplexwas targeted to the phytoene desaturase (PDS) endogenous gene and was designed using pssRNAit online tool provided by the *Zhao Bioinformatics Laboratory* based on a chinese spring wheat PDS mRNA sequence (acc. FJ517553.1) with the sequence 5′-CAUGUUGUGAAGACACCCGAG-3′ (sense) and 5′-CGGUGUCUUCACAACAUGGU-3′ (anti-sense).

To generate the scrambled siRNA duplex (scsiRNA) (5′-AGCCGGUACGAAUAGTGAGUC-3′ and 3′-UCGGCCAUGCUUAUCACUCAG-5′), the siRNA sense sequence was scrambled using the Genscript® online sequence scrambler; with rice being used as a reference genome. Both strands of the resultant sequence were tested for potential off-target sequence alignment with the *Ensembl Plants* database (*Triticum aestivum* and *Hordeum vulgare* genomes, genome assemblies TGACv1 and ASM32608v1), as well as the general *NCBI* database using BLASTn.

### RNA extraction, cDNA synthesis, and RT-qPCR analysis

The tip of the inoculated leaves (3–4 cm length by 1 cm width) was cut using scissors and placed into screwcap “tough tubes” (VWR) with 3 stainless steel beads (3 mm width) and immediately frozen in liquid nitrogen and stored at −80°C. To extract RNA the Nucleomag RNA 96 well plate (Macherey-Nagel) extraction kit was used, according to the manufacturers instructions with automation of extraction on a Biosprint 96 well plate robot (Qiagen). Purified RNA was quantified using UV-vis spectroscopy on a Nanodrop 8000 (ThermoFisher Scientific). cDNA was synthesized using a Superscript® VILO cDNA synthesis kit (ThermoFisher Scientific) according the manufacturers instructions with a total of 1.2 μg of RNA template per 20 μl reaction. RT-qPCR analysis was performed using SYBR-Green master mix (Life Technologies) with 40 ng of template cDNA per PCR reaction in 96 well plates. Each treatment and biological replicate was evaluated in three technical PCR replicates, with primers (500 nM forward and reverse) designed to amplify the target cDNA phytoene desaturase (PDS) and for three reference genes; ADP-ribosylation factor (ADP-RF), cell division control protein (CDC) and RNase L inhibitor protein (RLI) (see Table 1 for details). Thermocycling conditions were as follows: 95°C for 15 min, 40 cycles of 15 s of 95°C, 30 s of 60°C, and 30 s of 72°C, followed by a melting temperature series to evaluate amplicon content; 15 s at 95°C, 15 s at 60°C, and 15 s 95°C. All RT-qPCR was performed using an Applied Biosystems 7900HT RT-PCR machine and SDS 2.4 software. Reference genes were analyzed for stability by global averages of Ct-values (see Table S1) and the most stable was used for normalization of the target PDS gene expression level, based on the ΔΔCt method as amplification efficiencies were substantially similar (within <2%). Data was displayed as relative expression of PDS using 2^−ΔΔCt^ for conversion.

**Table 1:** **Primer-set Sequences for RT-qPCR**.

**Gene**	**Forward primer sequence (5′-3′)**	**Reverse primer sequence (5′-3′)**	**Source**
PDS	AAAGCAGGGTGTTCCTGAT	CATGGATAACTCGTCAGGGTTTA	Geneious software alignment with NCBI FJ517553.1
ADP-RF	TCTCATGGTTGGTCTCGATG	GGATGGTGGTGACGATCTCT	Giménez et al., 2011
CDC	CAGCTGCTGACTGAGATGGA	ATGTCTGGCCTGTTGGTAGC	
RLI	TTGAGCAACTCATGGACCAG	GCTTTCCAAGGCACAAACAT	

### Statistical analysis of RT-qPCR

Data was analyzed using ANOVA and unpaired *t*-test to detect significant differences between pairs of means, with calibration of all treatments to an *n* = 3 group of untreated controls. This was done to maintain a balanced system for ANOVA. Statistical significance was rated at the 95 and 99.99% confidence intervals, *p* < 0.05 and *p* < 0.0001 respectively. Two-way ANOVA was applied twice to evaluate factors affecting relative expression of PDS. The initial application evaluated variation of sample treatment (Control, TBPB, siRNA, Tat_2_, Tat_2_:siRNA, siRNA:TBPB, Tat_2_:siRNA:TBPB, scsiRNA, and Tat_2_:scsiRNA:TBPB), versus solvent used (ddH_2_O or maltose-mannitol). The second application of two way ANOVA gauged possible interaction between sample treatments (Control, siRNA, Tat_2_, Tat_2_:siRNA, and scsiRNA) with and without TBPB. In cases where no statistical significance between factors could be determined, data sets were combined and eight biological replicates were used per treatment in unpaired Student's *t*-test (see **Table 3**).

## Results

### Tat_2_ complexation with dsRNA

**Figure 1:**
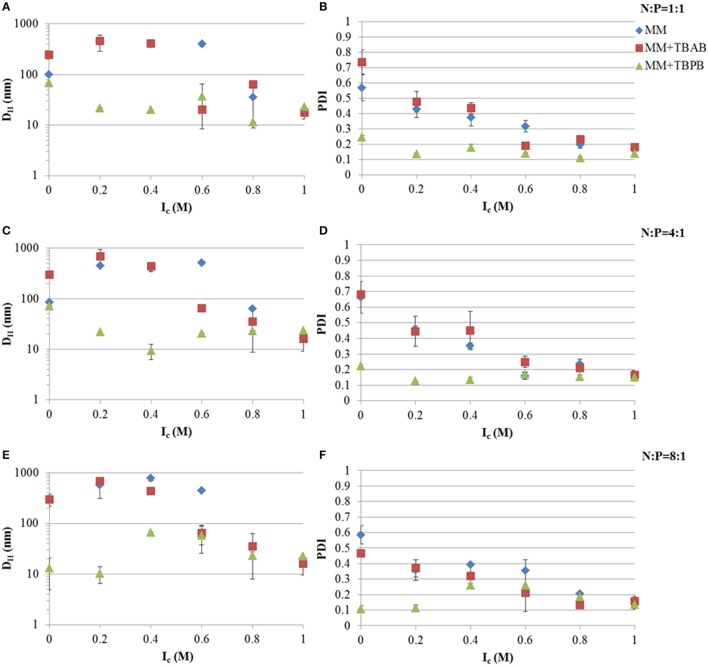
**Hydrodynamic diameters (D_**H**_) and PDI at different Tat_**2**_:dsRNA positive to negative molar charge ratios and ionic strength using CaCl_**2**_**. Charge ratios used were 1:1 **(A,B)**, 4:1 **(C,D)**, and 8:1 **(E,F)**. Data plotted are means ± s.d. (*n* = 3) using the CONTIN algorithm.

Hydrodynamic diameters and PDI of Tat_2_:dsRNA nano-complexes were measured using DLS. Tat_2_ complexation with 21-mer siRNA showed significant reduction in size and PDI upon the use of TBPB as an additional agent. It can be seen from **Figure 3** that at all charge ratios of Tat_2_:dsRNA, TBPB decreased the size of the nano-complexes but was most effective at the 8:1 Tat_2_:dsRNA N:P ratio, producing nano-complexes with a D_H_ of ~10 nm without addition of CaCl_2_ (see Figure 1). Generally it was observed that PDI correlates with size, lower size measurements correlating with lower PDI measurements. A PDI of ~0.1 indicates a strongly homogeneous size of the particles, and was only achieved using TBPB, with TBAB only improving complex formation at an I_c_ of 0.6 M before it was indistinguishable from the effect of CaCl_2_ alone. This trend was observed with CaCl_2_ throughout. It can be seen that as I_c_ increases, the addition of TBPB had a less dramatic effect that was indistinguishable from the effect of CaCl_2_ alone at I_c_ exceeding 0.6 M (see Figure 1).

### Tat_2_ complexation with DNA

**Figure 2:**
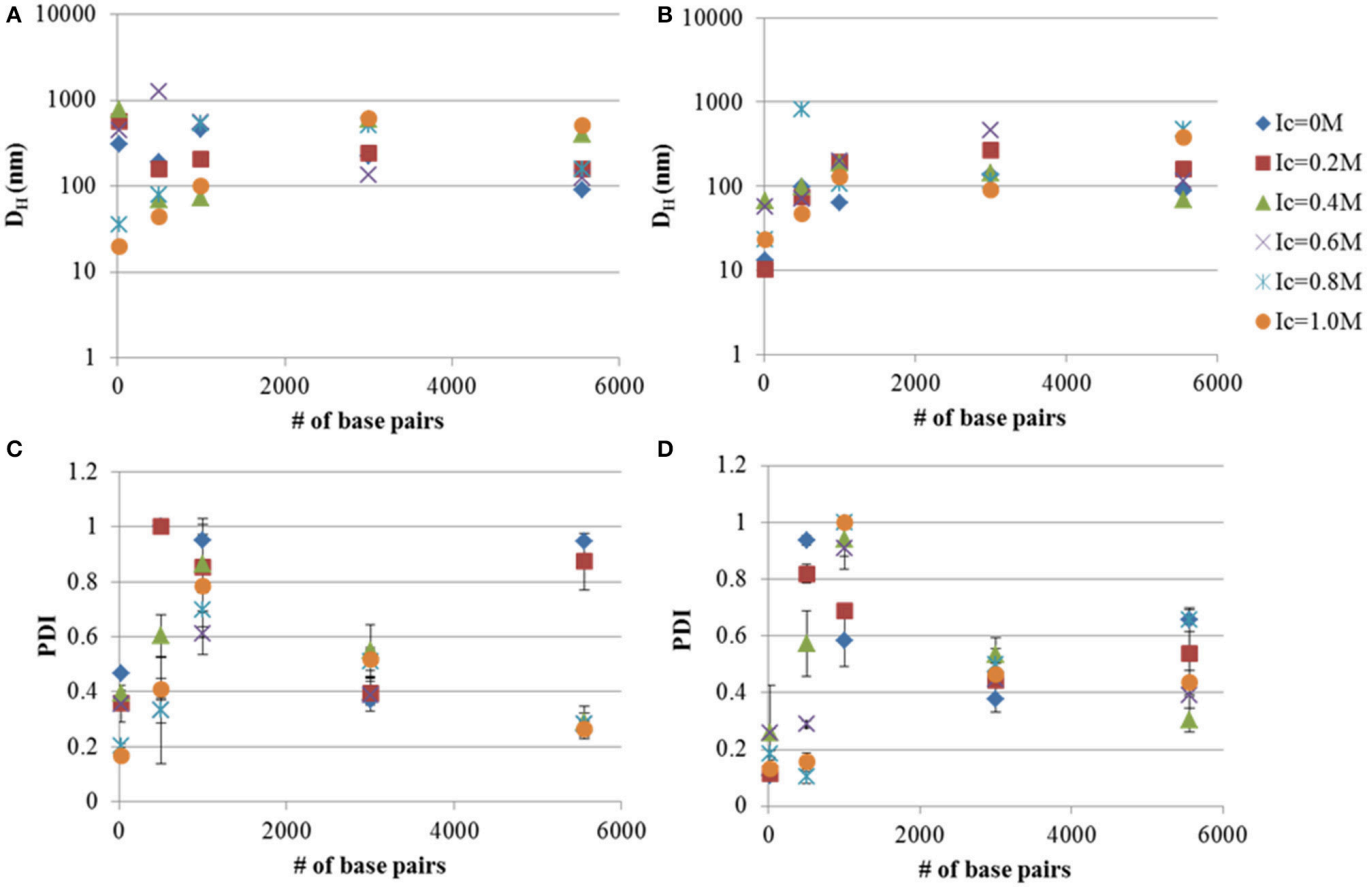
**Hydrodynamic diameters (D_**H**_) and PDI measurements of DNA-Tat_**2**_ nano-complexes in MM media with (B,D)** and without TBPB **(A,C)**. The 8:1 Tat_2_:dsRNA charge ratio is also shown for comparison. The relationship between the size of the nucleic acid in terms of # of base pairs and the size of the nano-complexes is shown. Data plotted are means ± s.d. (*n* = 3), evaluated using CONTIN.

Tat_2_:DNA nano-complex D_H_ were measured under different ionic strengths of CaCl_2_ and in the presence or absence of TBPB. The addition of TBPB to Tat_2_:DNA nano-complexes showed a noted lesser effect than that observed with dsRNA (see Figures 1, 2). The behavior of linear DNA did not strongly resemble the patterns observed with dsRNA. In the case of 0.5 kb, it was shown that TBPB had a weak effect on PDI, but reduced the size by ~ 50%, from 189.3 to 96.0 nm. This effect became more pronounced with an increase in ionic strength; however, at ionic strength of 0.8 the size increased to 811 nm, with reduction to a minimum in size at an I_c_ of 1.0 M of 46.95 nm. The PDI was found again to generally correlate inversely with I_c_ with a reduction to 0.155 at an I_c_ of 1.0 M.

In the case of the 1 kb DNA fragment (see Figure 2B), a true disparity in size and PDI can only be seen at the 0.0 M I_c_ limit, where size was at a minimum (62.6 nm) for the TBPB formulation and PDI was 0.538. A similar minimum was reached for formulation without TBPB at 0.4 M ionic strength with a size of 72.6 nm. The PDI was generally too high at all I_c_ higher than 0.0 M for appropriate analysis.

At 3 kb both MM and MM+TPBP produced nearly identical results in regards to PDI, while size was generally reduced to 90–120 nm under all I_c_ except for 0.2 M where MM and MM+TPBP formulations showed the same size particles (~ 250 nm) and 0.6 M, where the MM only formulation was shown to be significantly smaller (135.9 nm) and the MM+TBPB formula was substantially larger (461.5 nm). A complete divergence in size was noted after I_c_ 0.8 M.

For circular plasmid, the results show a close correlation in size under both MM and MM+TBPB condition, up to 0.2 M ionic strength and at 0.6 M. PDI was lowered substantially by addition of TBPB (a reduction of 0.3), but became smaller and more similar between MM and MM+TBPB formulations after an I_c_ of 0.4 M.

Generally it was shown that TBPB decreases particle size marginally, while often having a more significant effect on PDI up to <3 kb sized linear DNA and has less substantial effects at 3 kb linear DNA, in combination with Tat_2_. Increasing ionic strength generally decreases PDI, but is highly dependent on the size of the nucleic acid. Additionally, large sizes can be observed to occur at anomalous positions, particularly with 0.5 kb linear DNA, where the 0.8 M I_c_ increment was substantially larger than all other formulations (nearly eight-fold). As can be seen by comparing the TBPB positive samples to the TBPB lacking samples, the size of nano-complexes was more affected at the smaller size ranges, with the effect becoming very slight with circular plasmid, though all minimized sizes were found only in the TBPB containing formulations.

### Advanced size analysis

**Figure 3:**
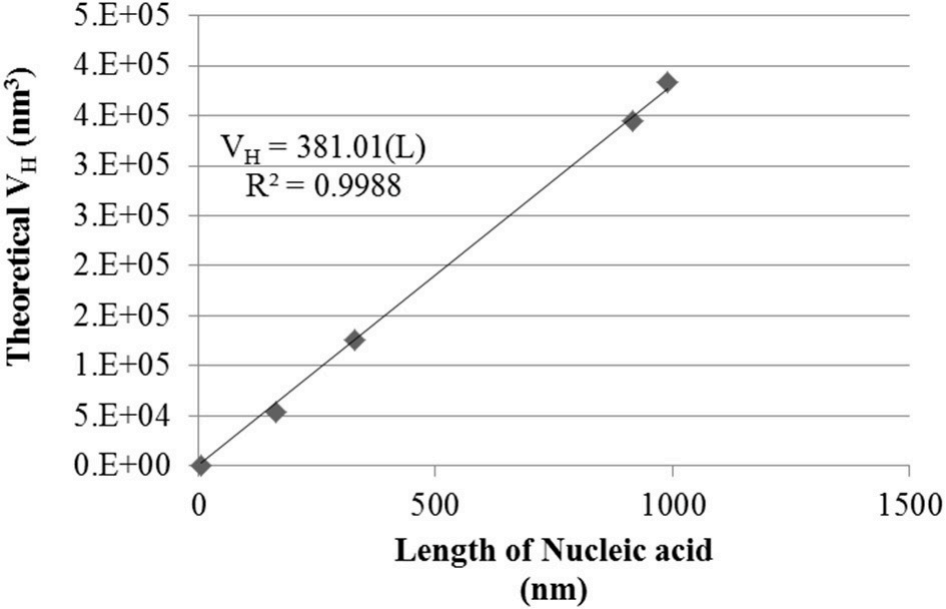
**Hydrodynamic Volume (V_**H**_) of size minimized nano-complexes of different lengths of nucleic acid**. Data shown is a conversion of the mean nano-complex size to hydrodynamic volume under the approximation of a sphere. The linear regression was forced through zero. *R*^2^ and regression equation are shown, where “L” is the length of the nucleic acid.

Theoretical number distribution based hydrodynamic volumes (V_H_) were calculated from the smallest Tat_2_ nano-complex sizes measured for each nucleic acid under their specific conditions (see Table 2). The volumes allow for a robust linear regression when related to the length of the nucleic acid (see Figure 3). It was also interesting that the approximation of the size of the circular plasmid to one half of its actual sequential length to account for secondary structure and circular shape, was well approximated to the regression shown (see Figure 3).

**Table 2:** **Minimally sized nano-complexes and formulations**.

**Nucleic acid: length & type (nt/nm)**	I_c_ CaCl_2_^*^ (M)	**Minimal D_H_ (nm)**	**Calculated V_H_ (nm^3^)**
dsRNA (21/6.93)	0.2	10.29	572.0
Linear dsDNA (500/165)	1.0	46.95	5.419 × 10^4^
Linear dsDNA (1,000/330)	0.0	62.30	1.266 × 10^5^
Circular plasmid (5.556/916.7)	1.0	86.96	3.443 × 10^5^
Linear dsDNA (3,000/990)	0.4	90.10	3.830 × 10^5^

* *All formulations required TBPB*.

By solving for the slope of the linear regression fit based on the equation V_H_ = 380.01(L), where the calculated slope of the regression appears to indicate a hydrodynamic volume increase of ~380 nm^3^/bp. Additionally, all the smallest measured nano-complexes (numerical values in Table 2) required the use of TBPB, though other formulations at high I_c_ were also found to give similar results (see Figure 2). It must be noted that this simplistic mathematical model does not take into account the entropic or enthalpic complexity of nucleic acid-cation interactions.

### Zeta-potential analysis

**Figure 4:**
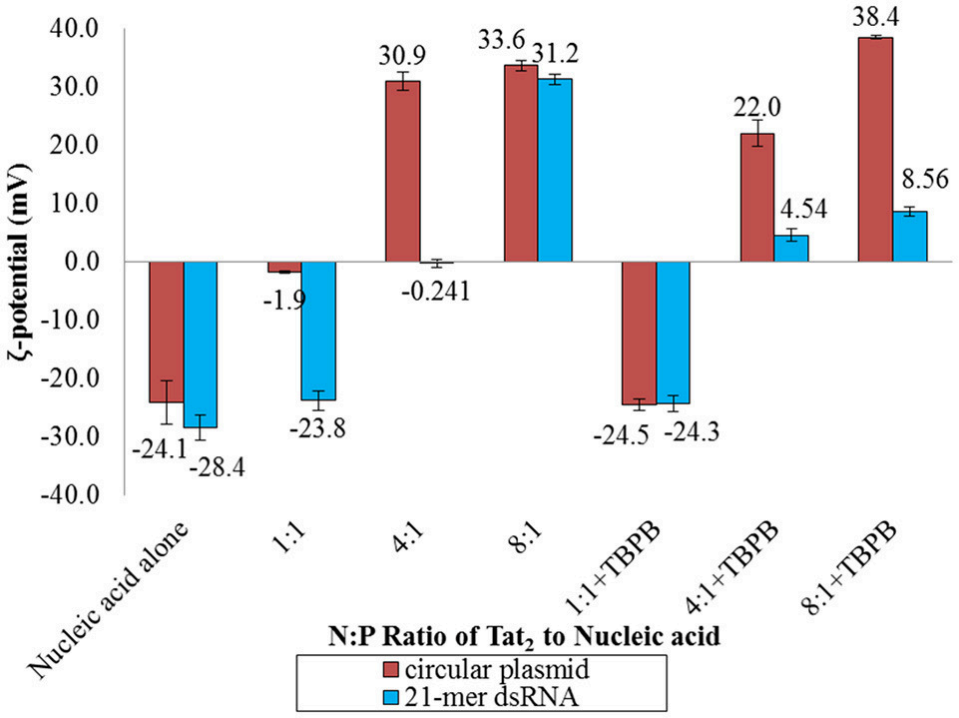
**Zeta (ζ) potential analysis of circular plasmid and dsRNA samples with and without TBPB in MM medium at the 1:1, 4:1, and 8:1 N:P ratios with Tat_**2**_**. Circular plasmid was preferred over the linear dsDNA due to the higher concentration required for zeta-potential measurements. Numbers displayed are the values of the bars in mV. Data displayed represent means ± s.d. calculated from triplicate measurements.

To determine the apparent charge character of the dsRNA nano-complexes and to determine if differences in how large dsDNA behaves in the presence of CPP and TBPB versus dsRNA as far as apparent charge character, zeta-potentials of Tat_2_:circular plasmid, and Tat_2_:dsRNA nano-complexes were measured at different N:P ratios, both with and without TBPB. At low N:P ratios (1:1 and 4:1), zeta-potential was greatly affected by TBPB, where circular plasmid was found to have virtually no change at the 1:1 N:P ratio level in the presence of TBPB while without TBPB a change of 22.2 mV occurred with the addition of Tat_2_. With dsRNA the addition of TBPB had no effect on zeta-potential at the 1:1 N:P ratio, with a measure of ~ −24 mV. At the 4:1 N:P ratio, the circular plasmid was overcharged to a zeta-potential of 30.9 without TBPB but significantly less at 22.0 mV with TBPB. The opposite trend was observed in the case of 21-mer siRNA, as the 4:1 ratio without TBPB resulted in a zeta-potential of neutralization at −0.241 mV, while with TBPB the potential was measured at 4.54 mV. Overcharging continued at the 8:1 ratio in the case of circular plasmid and dsRNA without TBPB to 30.9 and 33.6 mV respectively. Increase in zeta-potential was shown in the case of circular plasmid up to 38.4 mV in the presence of TBPB at the 8:1 N:P ratio. The increase in zeta-potential was comparatively marginal in the case of dsRNA at an average of 8.56 mV, an increase of only 4.02 mV compared to circular plasmid's increase of 16.4 mV (see Figure 4).

### RT-qPCR of inoculated triticale leaf samples

**Figure 5:**
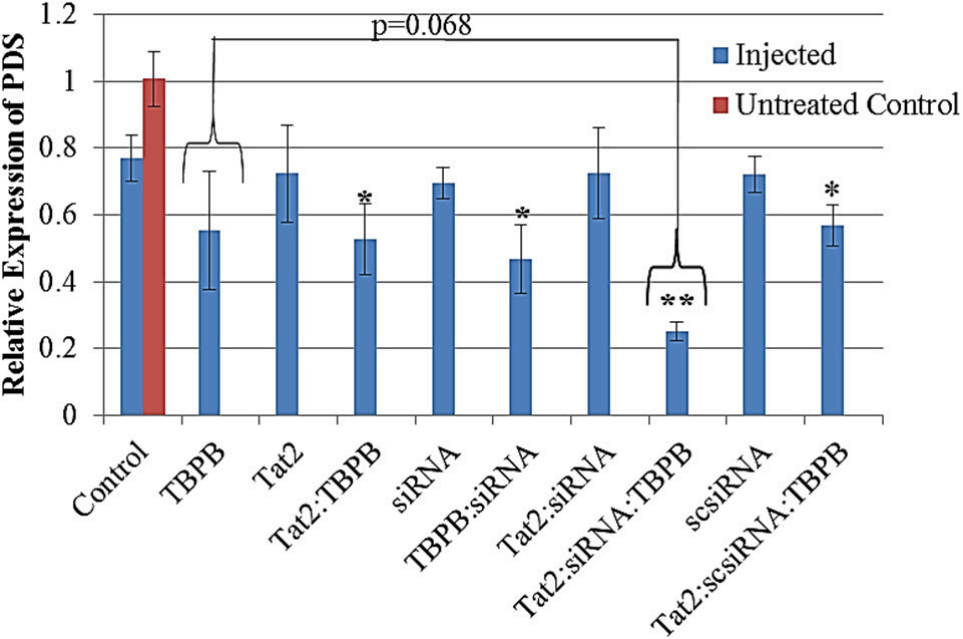
**Relative expression of PDS from seedling Triticale (Sunray) leaves converted from ΔΔCt values, normalized against ADP-RF**. All data was calibrated against an untreated control (Red bar, *n* = 3). A significance level of *p* = 0.00024 was found for the difference between treatments containing TBPB and those without using ANOVA. The (^*^) and (^**^) indicate a significance of *p* < 0.05 and *p* < 0.0001 between the designated treatment and the Control (solvent only), based on Student's *t*-test. Data displayed are means ± s.e. *n* = 8.

The expression level of the phytoene desaturase gene was evaluated using RT-qPCR, and converted to relative expression values using the 2^−ΔΔCt^ method. A reduction in relative transcript abundance by 20–30 % was observed in all samples that were treated by injection. Samples treated with TBPB, showed up to 45% reduction in expression with Tat_2_:TBPB, TBPB:siRNA, and Tat_2_:scsiRNA:TBPB, showing significances of *p* < 0.05. An approximately 75% reduction in PDS expression (*p* < 0.0001 compared to injected control) was only observed from the Tat_2_:siRNA:TBPB treatments (see Figure 5). Additionally, only the Tat_2_:siRNA:TBPB treatment was found to show a statistical difference from all other samples except for TBPB alone (*p* = 0.068) in unpaired comparisons using Student's *t*-test (see Table 3). Furthermore, it must also be noted that an especially high degree of statistical significance was noted between the Tat_2_:siRNA:TBPB treatment and the Tat_2_:scsiRNA:TBPB treatment at *p* = 4.21·10^−4^. This was noteworthy as these two treatments were the closest in composition, the only difference being the sequence of the RNA duplex (see Table 3). Still, it should be noted that the Tat_2_:scsiRNA:TBPB control was significantly different from the injection control and scsiRNA alone, which reflects the apparent effect of TBPB observed in the other treatments (see Table 3 and Figure 5).

**Table 3:** **Table of ***p***-Values for Unpaired Student's ***t***-Tests of RT-qPCR Samples**.

	**1**	**2**	**3**	**4**	**5**	**6**	**7**	**8**	**9**	**10**
1		0.145	0.395	0.041	0.198	0.016	0.394	3.22 × 10^−5^	0.304	0.024
2			0.235	0.452	0.233	0.343	0.227	0.068	0.196	0.471
3				0.147	0.426	0.086	0.496	6.18 × 10^−3^	0.495	0.173
4					0.091	0.347	0.135	0.018	0.067	0.376
5						0.036	0.416	2.97 × 10^−6^	0.354	0.063
6							0.077	0.036	0.025	0.211
7								4.47 × 10^−3^	0.489	0.156
8									7.63 × 10^−6^	4.21 × 10^−4^
9										0.040

*Key: 1, Control (solvent only); 2, TBPB; 3, Tat_2_; 4, Tat_2_:TBPB; 5, siRNA; 6, TBPB:siRNA; 7, Tat_2_:siRNA; 8, Tat_2_:siRNA:TBPB; 9, scsiRNA; 10, Tat_2_:scsiRNA:TBPB*.

ADP-RF expression was found to be the most stable reference for normalization, with CDC and RLI being far less stable. ADP-RF had a standard deviation amongst all tested samples of less than 1 Ct, while all other reference genes displayed standard deviations of greater than 1.3 Ct. Additionally it was found that for CDC and RLI samples that utilized TBPB had significantly lower Ct values than those without, with differences of up to 5 Ct, which represents an approximately 2^5^-fold difference in expression level. In the case of CDC this was a change of 22–24 Ct to 19–22 Ct, and in the case of RLI a change from 24–26 Ct to 20–22 Ct and as low as 18 Ct (see details in Table S1).

## Discussion

It must be understood that the principle size being measured is a hydrodynamic diameter, which reflects better the diffusion of particles in a medium rather than its actual size and shape. Its best application is in determining relative sizes of particles in different formulations and gauging PDI. It is with this in mind that these results must be evaluated.

In this study it has been shown with relatively high confidence that TBPB acts to help reduce both size and PDI of Tat_2_:nucleic acid nano-complexes, while TBAB showed little significant impact (see Figure 1). Because several sizes of nucleic acid were utilized, the mechanism by which this chemical acts may be elucidated to a limited degree. It may be hypothesized that it acts through the charge neutralization of phosphates on the backbone of nucleic acids, whereby compaction of the nucleic acids may be increased by the phosphonium cations. However, this cannot be the sole cause, as 21-mer siRNA has no extensive secondary structure with relatively low degrees of intermolecular freedom compared to longer nucleic acids. Small ~10 nm nano-complexes were formed with the addition of TBPB (Figure S1), while without it nano-complexes were 300 nm for the 8:1 N:P ratio of Tat_2_:dsRNA. It can be postulated that TBPB acts as a low concentration effective chaotropic, as shown previously (Harrison et al., 2006), breaking apart larger aggregates into smaller particles. This mechanism of action is substantiated as it is shown to only display substantial effects with linear dsDNA smaller than 3 kb. Since only the mass concentration of DNA was held stationary (1 ng/μl), the molarity of the DNA was reduced with increasing DNA length, meaning molar charge ratio was not likely the main contributor to size reduction but likely bulk molar ratio of particles to TBPB molecules.

Furthermore, the PDI measured for small nano-complexes was usually itself low (<0.3). Zeta-potential data also shows that the impact of TBPB was more significant with dsRNA, showing a substantially lower zeta-potential for dsRNA at the 8:1 N:P ratio (see Figure 4). This difference was nearly 30 mV, indicating that the apparent chaotropic effect was more significant in the case of dsRNA, and was able to disrupt but not abolish intermolecular interactions between Tat_2_ cationic amino acids and dsRNA phosphates. Interactions of tertiary phosphonium and ammonium cations of various types have been shown to interact strongly with DNA (Chatterjee and Moulik, 2005; Dehkordi et al., 2012), and similar interactions with dsRNA (Crnolatac et al., 2013). The previous work used phenyl substituted phosphoniums, and observed intercalative interactions with as well as electrostatic interactions, with similar organic groups forming hydrophobic interactions. While intercalative interactions are unlikely, hydrophobic shells and charge screening may have contributed to the formation of highly stabilized particles.

Calcium chloride concentration in terms of ionic strength was shown to reduce PDI in the case of dsRNA, 0.5 kb linear DNA, and circular plasmid. Calcium chloride has already been shown to reduce CPP:nucleic acid sizes, but was not tracked for effect on PDI (Khondee et al., 2011). In the present case it can be seen that CaCl_2_ did not significantly affect size except in the case of dsRNA. While it is known that divalent cations can complex, compact and/or precipitate DNA (Broekmans et al., 2016), it has also been shown that Ca^2+^ ions can bridge zwitterionic lipids with DNA to form liposomes (Antipina and Gurtovenko, 2015). It must be remembered that there exist two anionic glutamate residues in Tat_2_, and a similar adapter role for Ca^2+^ may explain the increased homogeneity, by occupying these residues and preventing aggregation. From this it can be inferred that CaCl_2_ has less of a chaotropic role in complex formation, and more of a stabilization role as it maintains the uniformity of the size of the particles, but does not actively reduce the size of the particles. It may do so through electrostatic screening of the nano-complexes as well.

This is the first time that a small molecule phosphonium salt has been used to reduce the size and PDI of CPP:nucleic acid nano-complexes, albeit with varying efficacies. It also illustrated the first formulation of CPP:nucleic acid nano-complexes in a dispersion medium high in maltose and mannitol. Polyphosphonium polymers have shown promise as low cytotoxicity transfection agents superior to their ammonium analogs, able to effectively complex nucleic acids at 1:1 molar charge ratios (Hemp et al., 2012a,b). It has been proposed that this highly efficient complexation is due to the higher localization of cationic charge on the phosphorus atom of the quaternary phosphonium versus the quaternary ammonium nitrogen atom (Wang et al., 2011). This may also contribute to the apparent chaotropic activity of TBPB.

It was also found that the minimal D_H_ of the nano-complexes, for each length of nucleic acid was related linearly to the volume of the nano-complex. The correlation was strong (see Figure 3), and showed that for each nm of nucleic acid there was an increase of ~380 nm^3^ in volume. It was also shown that the length of circular plasmid fit with the other data points when reduced to half its length to account for secondary structure (~916 vs. ~2,032 nm). This suggests that circular plasmid folds along a particular geometry that is functionally equivalent to a linear DNA half its length. It has been shown that nano-complexes of protamine and plasmid DNA form fasciculated structures, where DNA strands are located side by side after condensation (Motta et al., 2013). A similar structural formation may be the pattern observed with Tat_2_, but this is a tentative extrapolation.

All data being considered, these explanations do not account for the seemingly anomalous non-correlating points found during this study. Most notable would be the 0.5 kb sample at 0.8 M I_c_ CaCl_2_ and TBPB which measured at ~800 nm in diameter (see Figure 2). This was much larger, nearly tenfold, that of the non-TBPB formulation (~80 nm). No clear explanation for this exists within the scope of the methods used. However, this suggests that long linear DNA, expectedly, has more degrees of conformational freedom and may not always conform in the same manner when conditions change, due to more complex structural dynamics. Caution must be applied in these cases, as errors in measurement could also account for these differences.

The RT-qPCR data was able to show reasonably well that the use of TBPB in conjunction with Tat_2_ induced downregulation of the PDS gene with 21-mer siRNA. Evaluation of the statistical variation within each treatment shows that only the Tat_2_:siRNA:TBPB treatment was capable of manifesting a significant downregulation of the PDS gene by ~80% reduction in the relative abundance of the RNA transcripts. The only treatment that was not significantly different from the Tat_2_:siRNA:TBPB treatment was TBPB alone (*p* = 0.068). However, it can be seen that the distribution of the relative expression of PDS was higher in the TBPB alone treatment and crossed over significantly with most of the other controls, indicating a strong possibility of a false negative statistical significance at the p < 0.05 confidence interval. It could be predicted that the stress caused to leaves by injection and chemical administration may account for some of the reduction in relative PDS transcript abundance, the siRNA sequence was necessary to produce a tight sample distribution demonstrating transcript knockdown.

It was demonstrated that the silencing must have been systemic, as the portion of leaf tissue sampled was at the tip, while injections were performed at the base of the leaf. This implies that siRNA must have traveled via plasmodesmata or another vascular mechanism to induce silencing in the distally located tissue (Melnyk et al., 2011). It was also interesting to note that no significant difference in PDS gene transgene abundance was found between treatments using a maltose-mannitol solution as compared to ddH_2_O. This was surprising, because uptake of sugar solutions may be assisted through stimulation of phloem loading and unloading in plant vasculature (Lalonde et al., 2003; Peuke et al., 2015). This implies that the mechanism of siRNA nano-complex tissue dispersion and cell entry was not affected by the solvents used in these experiments, but instead was primarily affected by the formulation of the nano-complexes.

A possible reason why the Tat_2_:siRNA:TBPB treatment showed profound down regulation of PDS gene may be due to the reduced size of nano-complexes in that treatment. The nano-complex size between 10 and 20 nm was within the scope of the pores in plant cell walls. The pores of the cell wall of most plants do not exceed 20 nm (Chichiriccò and Poma, 2015) and since CPPs require contact with the plasma membrane to mediate import, smaller nano-complexes may demonstrate increased import efficiency. This is contrary to results in a prior study that show large nano-complexes exceeding 200 nm in diameter were able to transfect dicot plant cells with siRNA (Numata et al., 2014). However, these differences might be due to the structural and cellular differences between monocot and dicot leaves, such as veining structure and stomata arrangement.

Similar to prior results in plant cells, lower zeta-potentials also seemed to have enhanced efficiency. Additionally it may not be only import into the cells, but the subsequent release of the cargo which was enabled by the TBPB, as the reduced zeta-potential compared to that without TBPB, indicates less bound Tat_2_. This is further supported by the fact that the larger nano-complexes studied previously were also less bound by CPP (Lakshmanan et al., 2015).

Another possible contributing enhancer of transfection may be the potential of TBPB to slightly solubilize cellulose. It has been shown that with high concentrations (40% v/v) of its hydroxide variant (Abe et al., 2012) and with other similar ionic liquids (Kagimoto et al., 2006) they are capable of dissolving cellulose. Some slight dissolution of the cellulose in the leaf cell wall may have caused loosening of the cellulose allowing for easier entry of nano-complexes. Likely the explanation is a combination of all noted factors that allowed for the tentatively observed down-regulation.

The PDS gene was chosen due to its role in carotenoid synthesis, and previous use as a marker in virus-induced gene silencing (VIGS) in *Triticeae* species (Holzberg et al., 2002; Budak et al., 2015) where silencing was usually indicated by a photobleaching of the leaves. However, systemic silencing is likely necessary to observe photobleaching, therefore no such effect was observed in any of the treated plants up to 3 weeks after inoculation.

The primer sets chosen for all reference genes used, were previously shown to be stable across various species of the Triticeae tribe (Giménez et al., 2011), implying that this instability was well outside interspecies variation. However, even with the high degree of perturbation of a crucial cell division pathway, and RNA production regulation pathway, ADP-RF transcript levels remained relatively unaffected and appeared as the most stable reference gene. It was hence used for normalization of PDS expression levels. The other two CDC and RLI reference genes chosen, showed interesting effects in samples treated with TBPB. Both CDC and RLI transcript abundance increased in samples containing TBPB. One possible reason for the measured instability of these reference genes was because TBPB is related to long known growth retardant and regulator used in agriculture, known as phosphon (Balczewski et al., 2015). Also, it was reported that growth retardation of *Hordeum vulgare* and *Raphanus sativus* L. was caused by the similar ionic liquid salts triphenyl-hexadecylphosphonium iodide and triphenylmethyl-phosphonium iodide, which was observed as reduced growth height (Biczak et al., 2014). It is likely that these two genes were upregulated as a stress response, particularly CDC which plays a role in cellular division (Giménez et al., 2011), which was also upregulated the most, up to 2^5^ times the expression level of the treatments without TBPB (see Table S1). Due to this effect, it is likely that other chemicals similar to, but less toxic than TBPB, may need to be explored when applied to other genes of interest.

The work reported here opens the door to the use of similar formulations for dsRNA nano-complexes for gene silencing.Additionally, a strong mathematical relationship between nano-complex size and the length of the nucleic acid was discovered, resulting in a regression fit that indicated a 380 nm^3^ volume increase for every nm of nucleic acid for the CPP Tat_2_. It may be possible to extend this study to other peptides and determine whether substantially similar trends are observed. Finally this is the first report of nano-complexes of siRNA that may only include a small number or even a single siRNA molecule with a size between 10 and 15 nm.

## Author contributions

JP, Ideation, Experiment design and conduct, Data interpretation and analysis, MS prepration; PM, Ideation, Experiment design, Data interpretation, MS review and editing, corresponding author; AZ, Ideation, Experiment design; PH, Ideation, MS review; IK, Ideation, MS review; FE, Ideation, Experiment design, Data interpretation, MS review.

## Funding

This work was funded by Agriculture-Agri food Canada and KWS Germany.

### Conflict of interest statement

The authors declare that the research was conducted in the absence of any commercial or financial relationships that could be construed as a potential conflict of interest.
