# Supplementary material for: Tetrabutylphosphonium Bromide Reduces Size and Polydispersity Index of Tat2:siRNA Nano-Complexes for Triticale RNAi
Source: Front Mol Biosci. 2017 May 16;4:30. doi: 10.3389/fmolb.2017.00030 (PMC5432540; doi:10.3389/fmolb.2017.00030)
Supplement: Supplementary file 1 [file Table2.PDF]

Table S1: Ct value Analysis of Target (PDS) and Reference Genes from RT-qPCR of Triticale Leaves

MM=Maltose & Mannitol		Target Gene		Reference Genes					
		PDS		ADP-RF		CDC		RLI	
		**Water**	**MM**	**Water**	**MM**	**Water**	**MM**	**Water**	**MM**
**Control**	R1	25.97506	25.27929	23.69714	23.64619	24.89149	24.59786	27.55565	26.8921
	R2	24.09459	22.97824	22.32155	22.68338	22.50624	22.97363	25.32632	25.50647
	R3	24.89621	25.0647	23.08394	24.07086	23.60398	25.33332	26.07088	27.85261
**TBPB**	R1	23.8075	26.51612	23.5131	23.30975	19.80063	24.19953	24.34309	28.25299
	R2	25.19291	23.16497	22.32155	22.94547	21.94078	21.53990	25.55262	25.74529
	R3	24.78741	21.99968	23.08394	22.30749	20.68024	19.86071	25.14708	23.17134
**Tat_2**	R1	24.83638	24.98278	23.89314	23.05583	24.19563	23.67613	26.60516	26.46833
	R2	25.98721	24.48807	24.27212	24.70116	24.78406	24.94172	27.22974	26.97839
	R3	23.59364	22.32445	21.86751	21.99121	22.8027	22.26852	25.40135	24.36699
**Tat_2+TBPB**	R1	23.53142	24.62914	21.57527	22.68105	19.31091	21.34158	22.80724	25.29072
	R2	24.90273	22.93241	23.09212	22.63462	24.23861	20.6065	25.99182	24.88507
	R3	22.69374	21.69301	20.92079	21.20382	19.69844	22.30575	22.00518	18.81472
**siRNA**	R1	23.6736	23.10535	22.51141	21.20382	22.75313	22.30575	25.13446	24.36139
	R2	24.3346	24.86552	22.63000	22.78061	23.72576	23.80411	25.76133	26.07446
	R3	25.12562	22.92009	22.77035	20.993	24.643	20.16918	26.48736	23.5526
**TBPB+siRNA**	R1	23.95464	22.99029	21.96783	21.23991	19.47517	18.81622	23.77386	22.70514
	R2	27.65538	23.44015	24.70874	22.83608	23.18448	20.70164	26.23117	25.17861
	R3	22.77532	26.3533	21.36877	22.93817	18.8543	22.43513	23.76294	26.85483
**Tat_2+siRNA**	R1	23.69739	24.94946	22.79139	23.28964	23.56374	23.79088	26.56873	26.37328
	R2	23.36801	22.79671	22.14104	22.68721	22.49702	23.12447	25.34292	25.88212
	R3	26.69707	22.90538	23.55313	22.94949	24.65446	23.57826	26.85719	25.81883
**Tat_2+TBPB+siRNA**	R1	25.5705	24.87389	22.25095	21.67845	21.568	19.85769	26.00412	24.39663
	R2	25.13404	24.65055	22.59306	23.53069	20.51949	21.75225	25.47359	26.23117
	R3	25.40095	23.65055	23.52309	21.59999	21.22104	19.96935	26.25145	24.3767
**General average**		**24.65358**	**23.89809**	**22.76883**	**22.62324**	**22.29639**	**22.24792**	**25.48689**	**25.25128**
**General STDEV**		**1.21450**	**1.31722**	**0.92368**	**0.95134**	**1.94878**	**1.81074**	**1.34342**	**1.95342**

1
