# Supplementary figures and images for: Tetrabutylphosphonium Bromide Reduces Size and Polydispersity Index of Tat2:siRNA Nano-Complexes for Triticale RNAi
Source: Front Mol Biosci. 2017 May 16;4:30. doi: 10.3389/fmolb.2017.00030 (PMC5432540; doi:10.3389/fmolb.2017.00030)

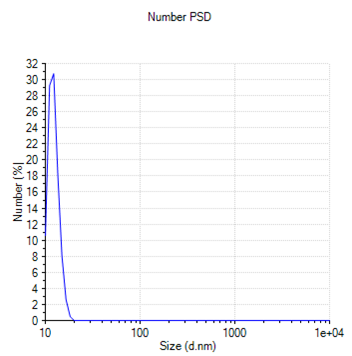

Supplement: Supplementary file 2 [file Image1.PNG]
